# Supplementary material for: Prostate Cancer Severity in Relation to Level of Food Processing
Source: Nutrients. 2023 Sep 16;15(18):4010. doi: 10.3390/nu15184010 (PMC10537278; doi:10.3390/nu15184010)
Supplement: Supplementary file 1 [file nutrients-15-04010-s001.zip › nutrients-2578823-supplementary.pdf]

Supplementary Table S1. Bi-variate logistic regression analysis testing the association of NOVA food group by level of processing and one additional confounding factor with prostate cancer severity.

	OR (95% CI)				
**Intermediate/high vs. low risk prostate cancers**	+ Age	+ BMI	+ Education	+ Smoking status	+ Physical activity
Unprocessed/minimally foods	0.35 (0.16, 0.77)	0.36 (0.16, 0.80)	0.35 (0.16, 0.76)	0.44 (0.19, 1.00)	0.39 (0.17, 0.89)
Processed culinary ingredients	0.60 (0.28, 1.29)	0.65 (0.30, 1.40)	0.68 (0.32, 1.46)	0.61 (0.27, 1.36)	0.67 (0.30, 1.49)
Processed foods	1.85 (0.87, 3.92)	1.94 (0.88, 4.24)	1.66 (0.76, 3.59)	1.35 (0.60, 3.04)	1.85 (0.83, 4.12)
UPFs	2.18 (1.03, 4.62)	2.24 (1.04, 4.82)	2.52 (1.16, 5.47)	1.86 (0.84, 4.11)	2.09 (0.95, 4.59)
